# Supplementary material for: Identification and Characterization of Novel Serpentoviruses in Viperid and Elapid Snakes
Source: Viruses. 2024 Sep 17;16(9):1477. doi: 10.3390/v16091477 (PMC11437479; doi:10.3390/v16091477)
Supplement: Supplementary file 1 [file viruses-16-01477-s001.zip › viruses-3156565-supplementary/Supplemental Data_Final.pdf]

Supplemental Table S1. Summary of novel serpentovirus genome contigs amplified by MiSeq Next Generation Sequencing. The base pair length and average coverage are shown for each contig. Genome regions present including 5′untraslated regions (5′), ORF1a (1A), ORF1b (1B), spike (S), ORF3 (3), ORF4 (4), matrix (M), nucleocapsid (N), ORF7 (7) and 3′ untranslated regions (3′) are also shown for each contig.

GenBank	Description	Base Pairs	Genes	Avg Coverage
PP898865	R19.197_RedTailBamboo_Fragment 1	26,201	5′, 1A, 1B, S	10,418.23
PP898866	R19.197_RedTailBamboo_ Fragment 2	226	S, 3	10,418.23
PP898867	R19.197_RedTailBamboo_ Fragment 3	4,289	3, 4, M, N, 7, 3′	11,582.23
PP898868	R19.1163_WhiteLipIslandViper	30,853	5′, 1A, 1B, S, 3, 4, M, N, 7, 3′	70.94
PP898869	R19.1809_WaglersViper_Virus1	28,707	5′, 1A, 1B, S, 3, 4, M	721.18
PP898870	R19.1809_WaglersViperVirus2	24,651	1A, 1B, S, 3, 4, M, N, 7, 3′	377.19
PP898871	R19.1985_GumprechtsPitViper_Frament 1	3,353	1A	1297.95
PP898872	R19.1985_GumprechtsPitViper_Frament 2	13,086	1A, 1B, S	1297.95
PP898873	R19.2243_RedTailBambooPitViper	29,814	1A, 1B, S, 3, 4, M, N, 7, 3′	2184.59

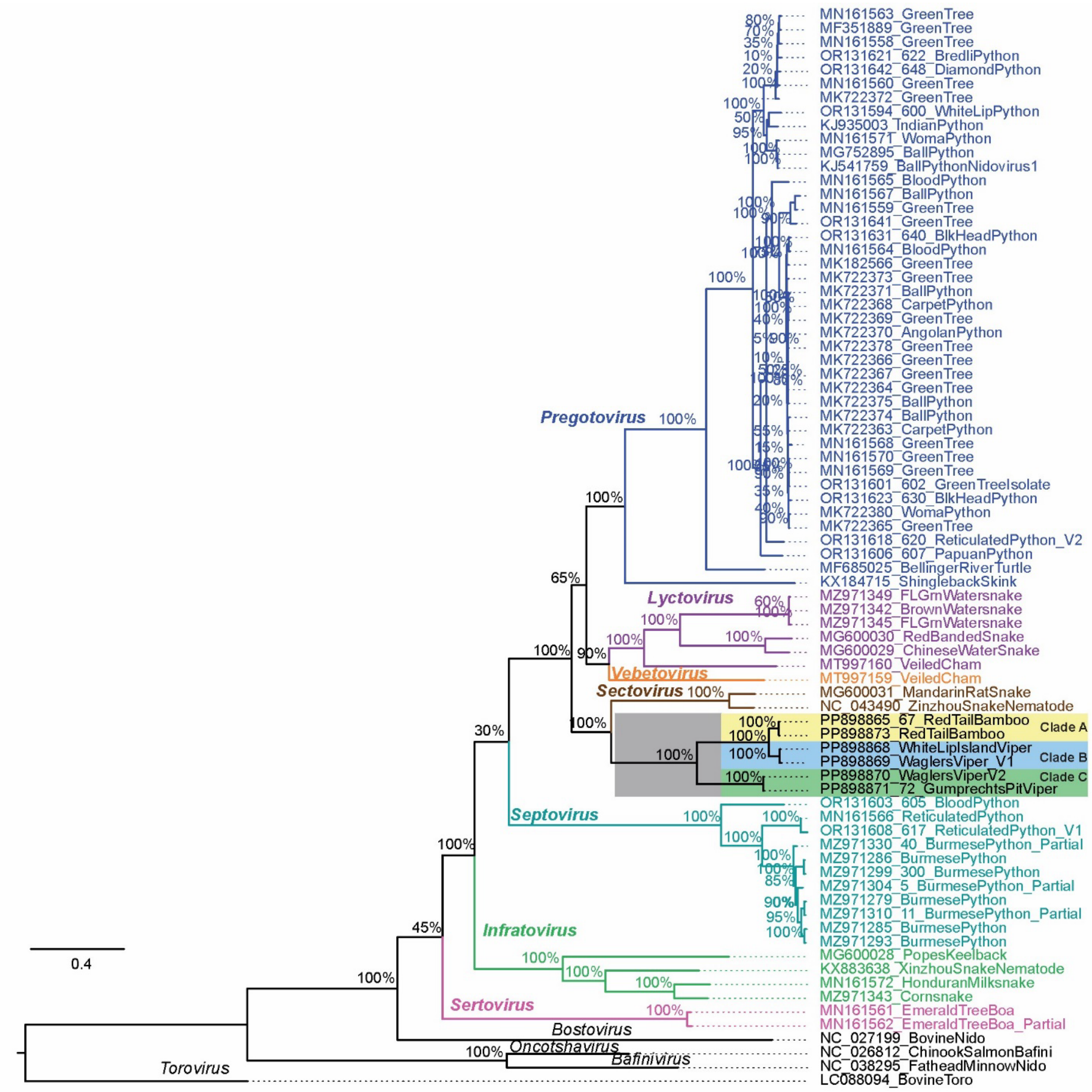

Supplemental Figure S1. Maximum likelihood model phylogenetic tree of serpentovirus open reading frame 1b (ORF1b: 1,197-2,938 aa, 3,212 positions) amino acid sequence with bootstrap values located at branch points. The divergent node containing six novel viper serpentoviruses are highlighted in gray and are further highlighted into three clades Clade A (yellow), Clade B (blue), and Clade C (green). The seven currently recognized serpentovirus genera are label and color coordinated to corresponding branches: *Pregotovirus* (blue), *Lyctovirus* (purple), *Vebetovirus* (orange), *Sectovirus* (brown), *Septovirus* (teal), *Infratovirus* (green), *Sertovirus* (pink), and outgroup or unclassified taxa (black). The bottom scale represents substitutions per site.

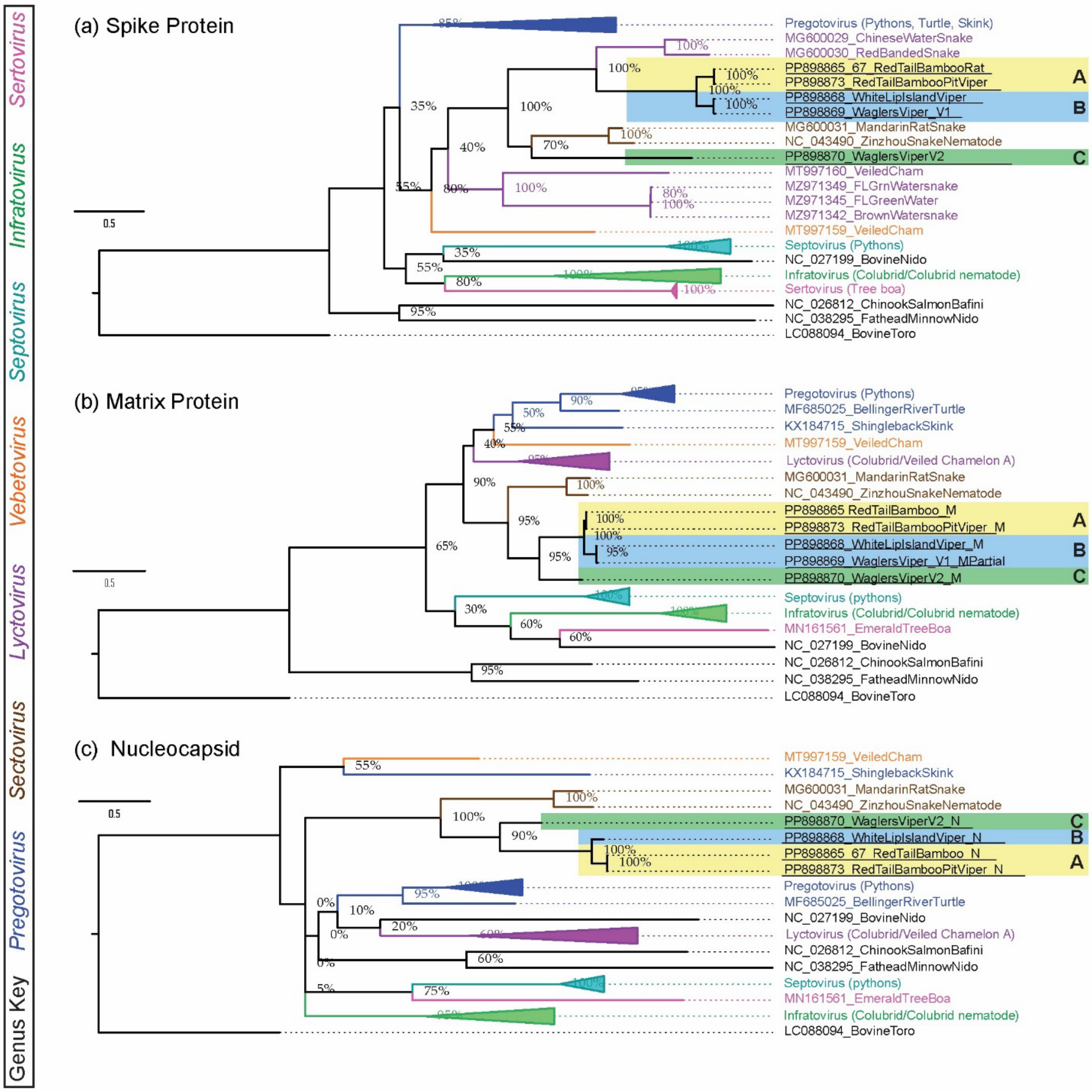

Supplemental Figure S2. Novel viper serpentovirus (a) spike [562-1,584 aa, 1762 positions], (b) matrix [115-281aa, 318 positions], and (c) nucleoprotein [107-246 aa, 347 positions] amino acid phylogenetic analysis via maximum likelihood models. Serpentoviral clades are collapsed where appropriate and bootstrap values are shown at branch points. Novel viper serpentoviruses are highlighted by clade (Clade A [yellow], Clade B [blue], and Clade C [green]) within the tree and labels are underlined. Existing serpentovirus genera are color-coordinated between trees: *Pregotovirus* (blue), *Lyctovirus* (purple), *Vebetovirus* (orange), *Sectovirus* (brown), *Septovirus* (teal), *Infratovirus* (green), *Sertovirus* (pink), and outgroup or unclassified taxa (black). The scales represent substitutions per site.
